# A Giant Solitary Adrenal Plasmacytoma in a Patient with HIV: A Rare Case Report and Review of the Literature

**DOI:** 10.1155/2021/6654437

**Published:** 2021-02-03

**Authors:** Mehdi Chennoufi, Ibrahim Boukhannous, Mohamed Mokhtari, Anouar El Moudane, Mohamed Irzi, Abdelghani Ouraghi, Issam Jandou, Achraf Miry, Ali Barki

**Affiliations:** ^1^Department of Urology, Mohamed VI University Hospital Center, Mohamed I University, Oujda, Morocco; ^2^Department of Pathology, Mohamed VI University Hospital Center, Mohamed I University, Oujda, Morocco

## Abstract

Solitary extramedullary plasmacytoma (EMP) involving the adrenal gland is an extremely rare malignancy. We report a case of a solitary adrenal plasmacytoma in an HIV-positive 50-year-old woman on antiretroviral therapy who presented with a rapidly progressing lumbar left masse. A CT scan objectified a locally advanced left adrenal mass measuring 135mm long axis. A biopsy was taken, and the histopathology with the immunohistochemical study objectified an adrenal gland plasmacytoma. The skeletal survey and the sternal suction biopsy did not show any abnormalities. The diagnosis of a solitary EMP of the adrenal gland was made. There are only 10 cases of solitary adrenal plasmacytoma with only one case associated with an HIV-positive patient reported in the literature. Therefore, this paper is aimed at presenting the second case of an HIV-positive patient diagnosed with solitary adrenal plasmacytoma.

## 1. Introduction

Extramedullary plasmacytoma (EMP) is a proliferation of monoclonal plasmatic soft tissue, without bone marrow involvement in patients with no clinical evidence of multiple myeloma (MM) [1]. It represents less than 4% of all plasma cell malignancies, of which 15% progress to MM and 80% are developing in the upper respiratory system and the head [2]. Any extraosseous organ can also be affected. Solitary adrenal gland plasmacytoma is extremely rare in an HIV-positive patient [3], unlike plasmablastic lymphomas [4]. It has been proven that the solitary EMP is touching this category of patients with younger age, atypical clinical course, and higher aggressivity. The diagnosis is a histopathological dilemma, while its management is still controversial. In the literature, there is no evidence of the superiority of laparoscopic retroperitoneal adrenalectomy compared with laparoscopic transperitoneal adrenalectomy in regard to the duration of surgery, operative blood loss, and conversion to open surgery, while late morbidity might be reduced following the retroperitoneal approach for small lesions (less than 6 cm to 7 cm) [5]. This case is proof that the plasma cell malignancies associated with a human immunodeficiency virus (HIV) could have an unusual localization, an unpredictable evolution, and a poor prognosis.

## 2. Case Report

A 50-year-old female patient who had been diagnosed with HIV 6 months previously under highly active antiretroviral therapy (HAART) was admitted for abdominal pain and mass syndrome of the left flank evolving for a month with a weight loss of 12 kg, in whom the clinical examination found a conscious patient, normotensive, with tenderness in the left flank, absence of clinical Cushing syndrome, or hyperandrogenism; the rest of the clinical examination is unremarkable.

**Figure 1:**
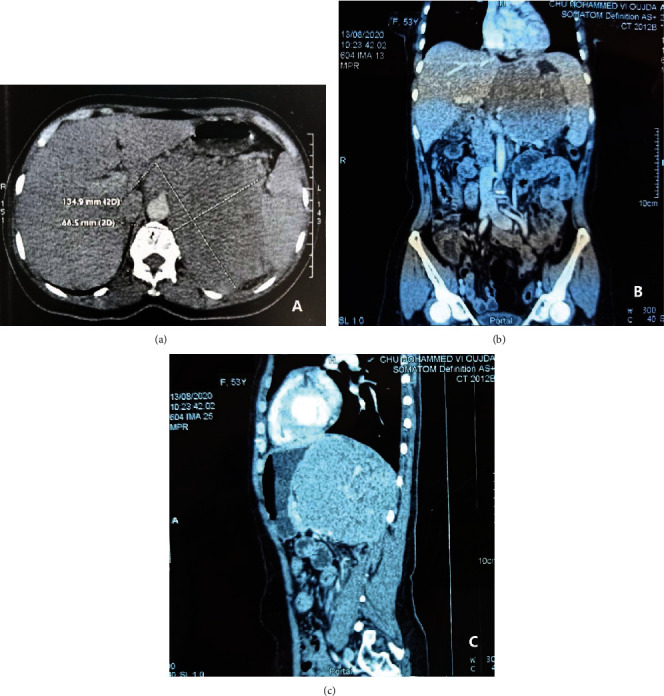
Abdominal CT scan: (a) axial, (b) frontal, and (c) sagittal images show the reports of the locally advanced left adrenal mass of 13.5 cm long axis.

The thoracic abdominopelvic scan (Figure 1) objectified a voluminous retroperitoneal tumor process centered on the left adrenal compartment badly limited irregular, containing zones of necrosis, measuring135 × 110 × 129 mm 10 HU of density, which infiltrates the great gastric curvature, the body and the tail of the pancreas without a dividing line, and the splenic vein which is laminated. Outside comes at intimate contact with a spleen includes the upper pole of the left kidney. Posteriorly, it arrives at the contact of the vertebral bodies D10, 11, and 12 and invades the left diaphragmatic pillar, the psoas muscle, and the square of the left lumbar region. Thus, it encompasses the celiac trunk and the renal artery over their entire circumference and the abdominal aorta over a circumference > 180° comes into contact with the superior mesenteric artery, and the common hepatic artery without a sign of invasion comes into contact with the inferior vena cava with a border of separation in places, associated with perilesional lymphadenopathy the largest 19 mm, left inferior lobar parenchymal nodule of 7 mm, and left supraclavicular lymphadenopathy the largest 13 mm.

Biological exploration revealed a negative endogenous hypersecretion of cortisol, negative urinary methoxylated metabolites, and no hypersecretion of androgens.

Creatinine was 5.56 mg/L, urea 0.27 g/L, natremia 139 mEq/L, kalmia 3.6 mEq/L, calcemia 100 mg/L, hemoglobin 12.3 g/dL, white blood cells 3110/mm^3^, and platelets 198,000/mm^3^, and the liver function test was unremarkable.

**Figure 2:**
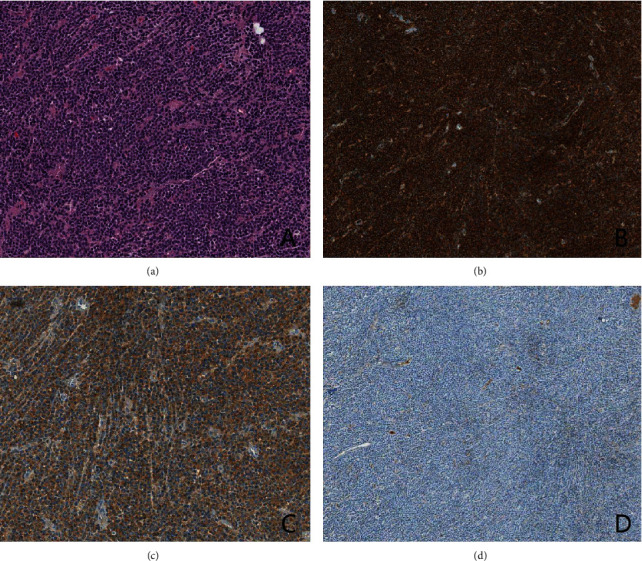
Microphotography showing sheets of atypical plasma cells (a). (HE, 100x). Immunohistochemical staining shows that the tumor cells were positive for CD138 (b), positive for lambda (c), and negative for kappa (d).

The patient was placed on level 2 analgesic treatment. A percutaneous biopsy of the mass was taken, showing a proliferation of atypical plasma cells. No intact tissue of the adrenal gland was found. Immunohistochemistry study revealed a negative expression of CD3 and CD20 excluding a plasmablastic lymphoma and a positive expression of CD138 and lambda light chains confirming the diagnosis of adrenal plasmacytoma (Figure 2).

Further serum investigation by immunoelectrophoresis of Bence-Jones proteins was made showing a high level of lambda light chains at 45.8 mg/L (normal level between 4 and 25.1 mg/L) with the normal level of kappa light chains at 6.2 mg/L (normal level between 5.2 and 22.17 mg/L). X-rays of the skeleton did not show lesions. A sternal suction biopsy did not show any plasmacytic infiltration. The diagnosis of a solitary adrenal plasmacytoma was established.

The patient was transferred to the oncology for chemotherapy and radiotherapy. But unfortunately, she died after 3 months of following up.

## 3. Discussion

Four categories of plasma cell malignancies exist: MM, plasma cell leukemia, solitary plasmacytoma of the bone, and EMP [4]. This latter is a rare malignant monoclonal plasma cell proliferation in patients with no clinical signs of MM. It represents less than 4% of all plasma cell malignancies [6] with 15% usually developing to MM [4]. 80% arise in the head, the oral cavity, and the upper respiratory tract, while 20% occur in the thyroid, breast, gastrointestinal tract, spleen, retroperitoneum, kidney, bladder, and testes. EMP is very rarely involving the adrenal gland [7].

Several retrospective studies revealed that the incidence of monoclonal gammopathy was from 3.8% to 26% in HIV-positive patients [8, 9] vs. 3.2% in the general population older than 50 years old [10]. The mechanisms of the high risk of plasma cell disorders in HIV patients are still poorly elaborated. However, two mechanisms were proposed to explain this situation: antigen stimulation and immunodeficiency. HIV viral antigens could stimulate the proliferation of B cells and the secretion of an immunoglobulin without the interference of T cells, most often due to chronic antigenic stimulation. Also, HIV can cause T cell dysfunction thus activating B cells without viral antigen stimulation [11].

The diagnosis is established on the findings of a plasma cell tumor in the exerted specimen or biopsy, the immunohistochemistry study, the immunological analysis of serum and urine, the absence of multiple myeloma on bone marrow suction biopsy, and the absence of skeletal lesions [3].

There are only 10 cases reported in the literature (Table 1). Most cases were reported in the Asian population. The age ranges from 26 to 77 years with a medium at 61 years. Sex ratio was 2.3 male/1 female. Our patient was a 50-year-old female. The right adrenal gland was more affected with three cases of bilateral tumors, and only one case of left adrenal plasmacytoma. The mean maximum dimension was 5.8 cm (range, 3.5 cm to 10 cm). Our case was the largest adrenal gland plasmacytoma reported to date with 13.5 cm in the long axis. Fujikata et al. [12] reported high levels of noradrenaline in the endocrinologic function with an elevation of monoclonal IgG-lambda at serum immunoelectrophoresis, contrasted with a monoclonality of IgG-kappa at immunohistochemical study, from where the diagnosis of extramedullary plasmacytoma independent of simultaneous benign M proteinemia was established. Ahmed et al. [13] reported bilateral regression of the adrenal tumors with calcification and fibrosis after two consecutive autologous hematopoietic stem cell transplantation. Cao et al. [14] reported the first case of extramedullary plasmacytoma involving the adrenal gland in an HIV-positive 35-year-old male patient. Of the six cases that have reported follow-up data on adrenal EMP, the follow-up ranging was from 12 to 72 months, with no recurrences.

Most HIV-positive patients are excluded from clinical trials. There are no guidelines for managing these specific patients. HAART is essential in the treatment of plasma cell disorders in HIV-positive patients. Yet, it is unclear how long the patients need to be on it before and during the course of chemotherapy. The role of RT is not clear unless there is a suspicion of residual disease [11]. The autologous hematopoietic stem cell transplantation is a possible alternative that needs more long-term studies.

## 4. Conclusion

Solitary EMP involving the adrenal gland in patients with HIV is extremely uncommon. The diagnosis is established on clinical, radiological, immunohistochemical, and histological data. The management is still controversial. More case reports and long-term-studies are needed to establish well-supported guidelines for these specific patients.

## Figures and Tables

**Table 1:** Solitary extramedullary adrenal gland plasmacytoma reported in the literature.

Study	Years	Country	Age	Gender	Side	Tumor size (cm)	Function	Treatment	HIV+	Serum protein electrophoresis	Kappa	Lambda	Follow-up (month)
Kahara [15]	2001	Japan	52	Male	Right	4	No	LCA	No	+	No	Specimen	12
Asahi [16]	2001	Japan	52	Male	Right	4	No	LCA+C +R	No	+	No	Specimen	
Fujikata [12]	2002	Japan	77	Male	Right	10	Yes	OA+nephrectomy+R		+	Specimen	Blood	12
Rogers [17]	2004	USA	75	Female	Right	3.5	No	LCA+R	No	—	No	Specimen	NP
Li [18]	2007	China	64	Female	Bilateral	6 right/4left	No	OA	No	—	No	Specimen	NP
Ahmed [13]	2009	Saudi Arabia	47	Male	Bilateral	8 right/8 left	No	C+autologous hematopoietic stem cell transplantation	No	+	Specimen + urine	No	47
Blanco [19]	2011	Spain	76	Female	Left	6	No	Adrenalectomy+R	No	NP	NP	NP	40
Cao [20]	2014	China	26	Male	Right	4.5	No	LCA	No	+	Specimen	No	72
Cao [14]	2016	China	35	Male	Right	3.5	No	LCA	Yes	+	No	Specimen	24
Townend/Graus [21]	2016	Australia	57	Male	Bilateral	5.5 right/9.5 left	No	Bilateral OA	No	+	Specimen+serum+urine	No	NP
Chennoufi	2020	Morocco	50	Female	Left	13.5	No	C+R	Yes	+	No	Specimen+blood	3

LCA: laparoscopic adrenalectomy; C: chemotherapy; R: radiotherapy; OA: open adrenalectomy; NP: no report.

## References

[1] World Health Organization (2002). *Pathology and Genetics of Tumours of Soft Tissue and Bone*.

[2] Galieni P., Cavo M., Pulsoni A. (2000). Clinical outcome of extramedullary plasmacytoma. *Haematologica*.

[3] de Camargo Moraes P., Thomaz L. A., Montalli V. A., Junqueira J. L. C., Ribeiro C. M. B., Oliveira L. B. (2016). Extramedullary Plasmacytoma Diagnosed in an HIV-Positive Patient by an Unusual Clinical Presentation. *Case Reports in Dentistry*.

[4] Joseph A. A., Pulimood S., Manipadam M. T., Viswabandya A., Sigamani E. (2016). Extramedullary plasmacytoma: an unusual neoplasm in a HIV-positive patient. *International Journal of STD & AIDS*.

[5] Arezzo A., Bullano A., Cochetti G. (2018). Transperitoneal versus retroperitoneal laparoscopic adrenalectomy for adrenal tumours in adults. *Cochrane Database of Systematic Reviews*.

[6] World Health Organization (2005). *Patology & Genetics—Head and Neck Tumors*.

[7] Herranz S., Sala M., Cervantes M., Sasal M., Soler A., Segura F. (2000). Neoplasia of plasma cells with atypical presentation and infection by the human immunodeficiency virus. A presentation of two cases. *American journal of hematology*.

[8] Dezube B. J., Aboulafia D. M., Pantanowitz L. (2004). Plasma cell disorders in HIVinfected patients: from benign gammopathy to multiple myeloma. *The AIDS reader*.

[9] Fiorino A. S., Atac B. (1997). Paraproteinemia, plasmacytoma, myeloma and HIV infection. *Leukemia*.

[10] Kyle R. A., Therneau T. M., Rajkumar S. V. (2006). Prevalence of monoclonal gammopathy of undetermined significance. *The New England Journal of Medicine*.

[11] Coker W. J., Jeter A., Schade H., Kang Y. (2013). Plasma cell disorders in HIV-infected patients: epidemiology and molecular mechanisms. *Biomarker Research*.

[12] Fujikata S., Tanji N., Aoki K., Ohoka H., Hojo N., Yokoyama M. (2002). Extramedullary plasmacytoma arising from an adrenal gland. *Urology*.

[13] Ahmed M., Al-ghamdi A., Al-omari M., Aljurf M., Al-kadhi Y. (2009). Autologous bone marrow transplanation for extramedullary plasmacytoma presenting as adrenal incidentaloma. *Annals of Saudi Medicine*.

[14] Cao D., Hu Y., Li L., Xiao W., Wei Q. (2016). Retroperitoneal laparoscopic management of a solitary extramedullary plasmacytoma associated with human immunodeficiency virus infection: a case report. *Oncology Letters*.

[15] Kahara T., Nagai Y., Yamashita H., Nohara E., Kobayashi K., Takamura T. (2001). Extramedullary plasmacytoma in the adrenal incidentaloma. *Clinical Endocrinology*.

[16] Asahi H., Iwasa Y., Komatsu K., Hirata A., Koshida K., Namiki M. (2001). A case of plasmacytoma involving adrenal gland. *Acta urologica Japonica*.

[17] Rogers C. G., Pinto P. A., Weir E. G. (2004). Extraosseous (extramedullary) plasmacytoma of the adrenal gland. *Archives of Pathology & Laboratory Medicine*.

[18] Li Y., Guo Y. K., Yang Z. G., Ma E. S., Min P. Q. (2007). Extramedullary plasmacytoma involving the bilateral adrenal glands on MR imaging. *Korean Journal of Radiology*.

[19] Blanco Antona F., Bahamonde Cabria S., Blanco Antona L., Marín Pérez-Tabernero A. (2011). Adrenal extramedullary plasmacytoma. *Cirugía Española*.

[20] Cao D., Li L., Liu L. (2014). Solitary extramedullary plasmacytoma of the adrenal gland: a rare case report with review of the literature. *International journal of clinical and experimental pathology*.

[21] Townend P. J., Kraus G., Coyle L., Nevell D., Engelsman A., Sidhu S. B. (2017). Bilateral extramedullary adrenal plasmacytoma: case report and review of the literature. *International Journal of Endocrine Oncology*.

